# Association of angiotensin II and receptors in peri-implantation endometrium with microvessel density and pregnancy outcomes of women with recurrent implantation failure after embryo transfer

**DOI:** 10.3389/fendo.2023.1206326

**Published:** 2023-08-29

**Authors:** Ruofan Qi, Tao Zhang, Yingying Zhang, Jacqueline Pui Wah Chung, Wen-Jui Yang, Chi Chiu Wang

**Affiliations:** ^1^ Department of Obstetrics and Gynaecology, The Chinese University of Hong Kong, Hong Kong, Hong Kong SAR, China; ^2^ Department of Infertility and Reproductive Medicine, Taiwan IVF Group Center, Hsinchu, Taiwan; ^3^ Department of Fertility and Reproductive Medicine, Ton-Yen General Hospital, Hsinchu, Taiwan; ^4^ Reproduction and Development Laboratory, Li Ka Shing Institute of Health Sciences, The Chinese University of Hong Kong, Hong Kong, Hong Kong SAR, China; ^5^ School of Biomedical Sciences, The Chinese University of Hong Kong, Hong Kong, Hong Kong SAR, China; ^6^ Chinese University of Hong Kong -Sichuan University Joint Laboratory in Reproductive Medicine, The Chinese University of Hong Kong, Hong Kong, Hong Kong SAR, China

**Keywords:** endometrium, receptivity, renin angiotensin system, angiogenesis, implantation

## Abstract

**Purpose:**

Investigate whether local angiotensin II (AngII) and its AngII type 1 and 2 receptors (AT1R, AT2R) in the endometrium are different and correlate with microvessel density in women with reproductive failure and pregnancy outcomes.

**Methods:**

Endometrium during the window of implantation from 40 women with recurrent miscarriage (RM) and 40 with recurrent implantation failure (RIF) were compared with 27 fertile women. Peri-implantation endometrium from 54 women prior to euploid embryo transfer were collected and compared in women with successful pregnancy and unsuccessful pregnancy.

**Results:**

Compared with fertile women, expression of AT2R was significantly lower, while AT1R/AT2R expression ratio was significantly higher in the stroma of the RIF group. Endometrium arteriole MVD was significantly lower and negatively correlated with the AT1R/AT2R expression ratio in the stroma of the RIF group. No significant differences and correlations were found in the RM group. Compared with the pregnancy group, expression of AT1R and AT2R were significantly lower in all compartments, but only AT1R/AT2R ratio was significantly higher in the stroma of the non-pregnancy group. Similarly, endometrium arteriole MVD was also significantly lower and negatively correlated with the AT1R/AT2R ratio in the stroma of the non-pregnancy group.

**Conclusion:**

Local renin-angiotensin system is dysregulated in peri-implantation endometrium and associated with abnormal angiogenesis in RIF and poor implantation outcome after embryo transfer.

## Introduction

Endometrial receptivity is a major focus in the field of reproductive medicine. Robust angiogenesis in the endometrium is essential for the success of implantation and early pregnancy (1, 2), which is usually achieved by vasodilatation and remodeling of the endometrial arteries (3). Inadequate and excessive vascularity in the endometrium may influence endometrial receptivity thus resulting in implantation failure and miscarriage (4). Numerous studies were conducted to understand the relationship between endometrial blood supply and endometrial receptivity (5–8). Currently, there are two main methods to assess endometrial vascularity, one is the conventional method using immunohistochemical analysis in endometrial biopsy to evaluate microvessel density (MVD), and the other is a relatively new method using 3-dimensional power Doppler ultrasonography (3-DPD) assess the endometrial blood flow strength. However, the histological morphometrics in the endometrium may not necessarily correlate well with the ultrasound findings (9). This could be due to immunohistochemistry assessing the microvasculature, while 3-DPD can only detect macrovasculature (10).

The renin-angiotensin system (RAS) is a classic endocrine system, which has been known for its crucial role in maintaining systematic blood pressure and cardiovascular homeostasis. RAS consists of a cascade of peptides; after being cleaved from angiotensinogen by renin, Angiotensin I (Ang I) is converted by angiotensin-converting enzyme (ACE) to the most bioactive peptide Angiotensin II (Ang II). Ang II has regulatory effects mainly on angiogenesis and vasculogenesis through its two receptors, Ang II type 1 receptor (AT1R) and type 2 receptor (AT2R) (11–14). Both are vasomotor, while AT1R mediates vasoconstriction, AT2R mediates vasodilation (15). The balance between AT1R and AT2R may work in concert to influence the systematic vascular tone, perfusion pressure, and thus blood flow and tissue function (16). In the pathophysiologic state, the balance of AT1R and AT2R is highly controlled (17, 18).

Local RAS has been identified in the reproductive system, indicating its potential biological effects on reproductive functions (19). In women, various components of RAS are expressed in the ovary, uterus, and placenta (20–23). This includes Ang II in particular, which regulates oocyte maturation, ovulation, and corpus luteum under stimulation of gonadotropins and female sex hormones (24–26). Ang II and its receptors are also important in early embryonic development, where RAS is reported to participate in the dialogue between mother and developing embryo in rat and bovine (27, 28). In addition, activation of Ang II and its receptors leads to potent induction of vascular endothelial growth factor (VEGF) (29) and establishment of circulation in the placenta and decidua to maintain pregnancy (30–32). However, systematic investigation of the local RAS system in endometrium and its potential role in endometrium receptivity and implantation in humans is still very limited.

Recurrent miscarriage (RM) and recurrent implantation failure (RIF) are two major hurdles in reproductive medicine, whether they share any common or have different endometrial defects remains controversial (33, 34). Some suggest RM and RIF may share similar etiology (35–37), but cytokine profiling showed different results between the endometrium from women with RM and RIF (38). It was hypothesized that women with unexplained RM could be over-receptive contrary to that of women with RIF, which means the endometrium of RM is less likely to identify the abnormal embryos and prevent them from implantation, hence pregnancy loss after implantation (39, 40). Whether endometrium vasculature is different between RM and RIF and associated with endometrium angiogenesis, in this study our first aim was to investigate the localization and expression of Ang ll and its receptors AT1R and AT2R and correlate with arteriole and capillary in peri-implantation endometrium among women with RM or RIF in natural cycle with fertile women. On the other hand, good-quality embryos are a pre-requisite for successful embryo implantation. Recent technology of preimplantation genetic testing (PGT) allows the detection of aneuploid embryos, and ensures only euploid embryos are transferred and also excludes confounding factors from the embryo. However the IVF outcomes are still not significantly improved and the endometrium factor remained to be evaluated. Therefore, in another cohort of this study we included women in a mock cycle and compared endometrium RAS between women who did and did not conceive prior to PGT selected frozen embryo transfer (FET). Our hypothesis was that derangement of local RAS in peri-implantation endometrium may be associated with abnormal angiogenesis and poor IVF outcome in women with reproductive failure.

## Materials and methods

### Subjects

In the first cohort study, a total of 107 women, including 27 fertile controls, 40 with unexplained RM, and 40 with unexplained RIF in the natural cycle were recruited and LH surge was identified by daily urine dipstick test from day 9 of the menstrual cycle onwards. Fertile controls were defined as women who had at least one live birth without any major pregnancy complications, such as pregnancy-induced hypertension, within two years. RM was defined as women who had a history of ≥3 consecutive miscarriages before gestational week 20, while RIF was defined as women who failed to achieve a clinical pregnancy after transferring at least 4 morphologically good-quality embryos in a minimum of 3 cycles (41, 42). The subjects were recruited from the Prince of Wales Hospital, The Chinese University of Hong Kong in Hong Kong.

In the second cohort study, a total of 54 women undergoing FET after PGT were recruited. All these women were in a mock hormone replacement treatment (HRT) cycle, and oral estradiol valerate 6 mg daily was administered from the second day of the menstrual cycle. Ultrasound examination was performed to measure endometrial thickness, and vaginal progesterone (utrogestan, 600 mg per day) was administered if the thickness reached more than 8 mm and no peri-ovulatory leading follicle was present. In the following HRT cycle, only one euploid blastocyst confirmed by PGT was transferred. The pregnancy group was defined based on positive serum β-hCG measurement at 9 days after ET, a viable intrauterine fetus in transvaginal ultrasonography at 23 days after ET, and the pregnancy continued till at least 20 weeks gestation. Non-pregnancy group was defined as having negative serum β-hCG (<5 mIU/L) at 9 days and no viable fetus at 23 days after ET. We also excluded those women who conceived but later miscarried. The subjects were recruited from Ton‐Yen General Hospital and Taiwan IVF Group Center in Taiwan.

### Inclusion and exclusion criteria

Inclusion criteria included age between 25 and 45 years old and women with regular menstrual periods (25–35 days) and normal BMI. Exclusion criteria included women with endometriosis, hydrosalpinx, structural uterine abnormalities, steroid hormone treatment within 3 months of recruitment, known causes of RM and RIF, including antiphospholipid syndrome, thrombophilia, abnormal thyroid function tests, and any parental chromosomal abnormalities.

### Endometrial biopsy

In the first cohort study, the endometrial biopsies were precisely timed at LH surge (LH+7 day) in a natural menstrual cycle. In the second cohort study, endometrial biopsies were precisely timed at 5 days after progesterone treatment (P+5 day) in a mock HRT cycle. All endometrial samples were collected by using a Pipelle sampler (Prodimed, France). Then all the collected samples were rinsed and cleaned with sterile PBS, and then put into 10% neutral‐buffered formalin for overnight fixation before being embedded into paraffin wax for immunohistochemistry staining. The histological endometrium dating was confirmed by standard criteria of Noyes et al. (43) under hematoxylin and eosin staining showing a mid-secretory stage with distended glands and stromal edema.

### Immunohistochemistry

After embedding in paraffin wax, endometrial tissues were cut in 3.5 μm sections consecutively and mounted onto 3-aminopropyl-triethoxysilane (Sigma-Aldrich, St. Louis) coated slides. The serial sections were dewaxed, stained, and examined under the standard protocol and conditions to minimize the variations. The sections were dewaxed in xylene, rehydrated through descending alcohols to phosphate-buffered saline (PBS) (pH 7.6), and quenched in 3% hydrogen peroxide in methanol for 20 min. After washing, the antigen was retrieved in sodium citrate buffer (pH 6.0) in a microwave oven. The buffer was pre-heated in the microwave oven until boiling, after that the slides were cooled down for at least 20 min. The slides were washed in PBS and incubated in blocking solution (5% goat serum albumin in PBS) for 1 h at room temperature, and incubated overnight at 4°C with primary antibody, either rabbit anti-human Ang II polyclonal antibody (PA5-33339, Thermo Fisher, US) at 1:400 dilution, rabbit anti-human AT1R polyclonal antibody (AAR-011, Alomone Labs, Israel) at 1:1500 dilution, rabbit anti-human AT2R antibody (ab19134, AbCam, UK) at 1:150 dilution, or rabbit polyclonal anti-human vWF antibody (A0082, DAKO, Japan) at 1:1000 dilution. The sections incubated with blocking solution with no primary antibody were used as the negative control. After incubation, slides were washed in Tween‐20 in PBS and incubated with secondary antibody (goat anti-rabbit) for 1 h. The immunoreactive binding was visualized by incubation with peroxidase substrate DAB (3.3-diaminobenzidene tetrahydrochloride (DAB, Dako). The slides were then washed in distilled water and counterstained with 20% hematoxylin solution for 10 min and dehydrated through alcohols, cleared in xylene, and mounted in DPX medium (Sigma-Aldrich, St. Louis).

### Image analysis

All immunostaining results were under a standard microscopic examination protocol in a blinded manner. The first field was selected from the upper left of each section including luminal epithelium (LE) and glandular epithelium (GE) of the endometrium under the magnification at ×400. The subsequent field was then obtained by moving to another field just next to the last field. This was repeated until 5 fields with LE and GE, and also stroma (ST) of the endometrium in every field were captured. Localization of each protein in each compartment of the endometrium was examined and their expression levels were quantified by H score using inForm 2.4 Software (Perkin‐Elmer/Caliper Life Science, US). The intensity of the immunohistochemical signals in each compartment was classified into 0, 1+, 2+, and 3+, and then the percentages of each positively stained cell were computed as absent (0%), weak (30%), moderate (50%), or strong (70%). The average of the measurements from at least 5 sections were calculated for comparison. Inter- and intra-observer variability were 3.42% and 5.61%.

### Micro-vessel density

The endometrial sections were scanned to identify the hot spots of microvessels in the ST of the endometrium, representing the areas of high vascularization. AT2R, but not AT1R, mostly stained positive in arteriole endothelium with a clear cuff of one to two layers of smooth muscle. While vWF is mainly expressed in capillary endothelium with thin-walled vessels consisting of a single layer of endothelial cells without any smooth muscle cells (44). Total numbers of arteriole and capillary micro-vessels in the stroma were separately counted in 5 highly vascularized areas in order to avoid selection bias and minimize the variation and then calculated as average endometrium arteriole and capillary MVDs per square millimeter, vessels/mm^2^ (45, 46). The results were correlated with the non-microvessel AT2R-expressed stroma cells.

### Statistics

Data were analyzed using SPSS, version 26.0 (SPSS, Inc). The data distribution was checked by the Shapiro-Wilk test. Quantitative data were expressed as mean ± standard error of the mean (SEM) if normal distribution, and median and inter-quartile range if skewed distribution. AT1R/AT2R expression ratio was calculated based on the expression levels in each compartment quantified by H score. Differences between groups were assessed by independent t-test, One-way ANOVA, or non-parametric Kruskal-WaIIis test where appropriate. Correlation was performed by non-parametric Spearman’s rho and Kendall’s tau- b statistics. The sample size was calculated based on our pilot study with a power of 0.8 and type 2 error <0.05.

## Results

### Demographic characteristics

Detailed demographic characteristics of the women who participated in each cohort are summarized in **Table 1**. Age and BMI were not significantly different amongst groups in each cohort, but age was significantly higher in the second cohort with a mock HRT cycle. In the first cohort with the natural cycle, the numbers of previous pregnancies and live births were significantly lower and the number of failed cycles was significantly higher in the RIF group, while the numbers of previous miscarriages were significantly higher in the RM group. In the second cohort with mock HRT cycle, numbers of previous pregnancies, live births, previous miscarriages, and failed cycles were not statistically significant. No significant difference was found between women in a natural cycle and in an HRT cycle.

**Table 1:** Demographic characteristics.

Parameter	Natural cycle					Mock HRT cycle				P value^c^
	Overall (n=107)	Control (n=27)	RM (n=40)	RIF (n=40)	P value^a^	Overall (n=54)	Pregnancy (n=31)	Nonpregnancy (n=23)	P value^b^	
Age (y)	34.4 ± 2.7	32.5 ± 3.0	34.8 ± 2.4	35.9 ± 2.6	0.038	38.7 ± 5.3	37.5 ± 6.1	39.9 ± 4.9	0.210	0.081
BMI (kg/m2)	21.8 ± 2.4	21.0 ± 1.9	22.6 ± 2.9	21.7 ± 2.6	0.360	22.6 ± 1.2	22.3 ± 1.5	22.9 ± 1.1	0.350	0.237
No. of previous pregnancies										
0	33/107 (31%)	0/27 (0%)	0/40 (0%)	33/40(83%)	<0.001	13/54 (24%)	7/31 (23%)	6/23 (26%)	0.766	0.568
1	22/107 (21%)	15/27 (56%)	0/40 (0%)	7/40 (17%)		14/54 (26%)	9/31 (29%)	5/23 (22%)		
≥2	52/107 (48%)	12/27 (44%)	40/40 (100%)	0/40 (0%)		27/54 (50%)	15/31 (48%)	12/23 (52%)		
No. of live births										
0	74/107 (69%)	0/27 (0%)	36/40 (90%)	38/40(95%)	<0.001	21/54 (39%)	12/31 (39%)	9/23 (39%)	0.975	0.108
1	30/107 (28%)	24/27 (89%)	4/40 (10%)	2/40 (5%)		22/54 (41%)	13/31 (42%)	9/23 (39%)		
≥2	3/107 (3%)	3/27 (11.1%)	0/40 (0%)	0/40 (0%)		11/54 (20%)	6/31 (19%)	5/23 (22%)		
No. of previous miscarriages										
0	61/107 (57%)	27/27 (100%)	0/40 (0%)	34/40(85%)	<0.001	28/54 (52%)	16/31 (51%)	12/23 (52%)	0.967	0.237
1	6/107 (6%)	0/20 (0%)	0/40 (0%)	6/40 (15%)		13/54 (24%)	9/31 (29%)	4/23 (18%)		
2	0/107 (0%)	0/20 (0%)	0/40 (0%)	0/40 (0%)		4/54 (7%)	3/31 (10%)	1/23 (4%)		
≥3	40/107 (37%)	0/20 (0%)	40/40 (100%)	0/40 (0%)		9/54 (17%)	3/31 (10%)	6/23 (26%)		
No. of failed cycles										
0	67/107 (63%)	27/27 (100%)	40/40 (100%)	0/40 (0%)	<0.001	5/54 (9%)	5/31 (16.1%)	0/31 (0%)	0.053	0.065
1	0/107 (0%)	0/40 (0%)	0/40 (0%)	0/40 (0%)		16/54 (30%)	11/31 (35.5%)	5/31 (16.1%)		
2	0/107 (0%)	0/40 (0%)	0/40 (0%)	0/40 (0%)		15/54 (28%)	7/31 (22.6%)	8/31 (25.8%)		
≥3	40/107 (37%)	0/40 (0%)	0/40 (0%)	40/40(100%)		18/54 (33%)	8/31 (25.8%)	10/31 (32.3%)		

The data are presented as mean ± SEM or n/N (%).

BMI, body mass index; Control, normal fertile control; RM, recurrent miscarriage; RIF, recurrent implantation failure; HRT, hormonal replacement treatment.

a One Way ANOVA or Chi-square test to compare among Control, RM, and RIF groups.

b t-test or Chi-square test to compare between Pregnancy and non-pregnancy groups.

c t-test or Chi-square test to compare between the natural cycle and Mock HRT cycle.

### Comparison among fertile controls, RM, and RIF

**Figure 1:**
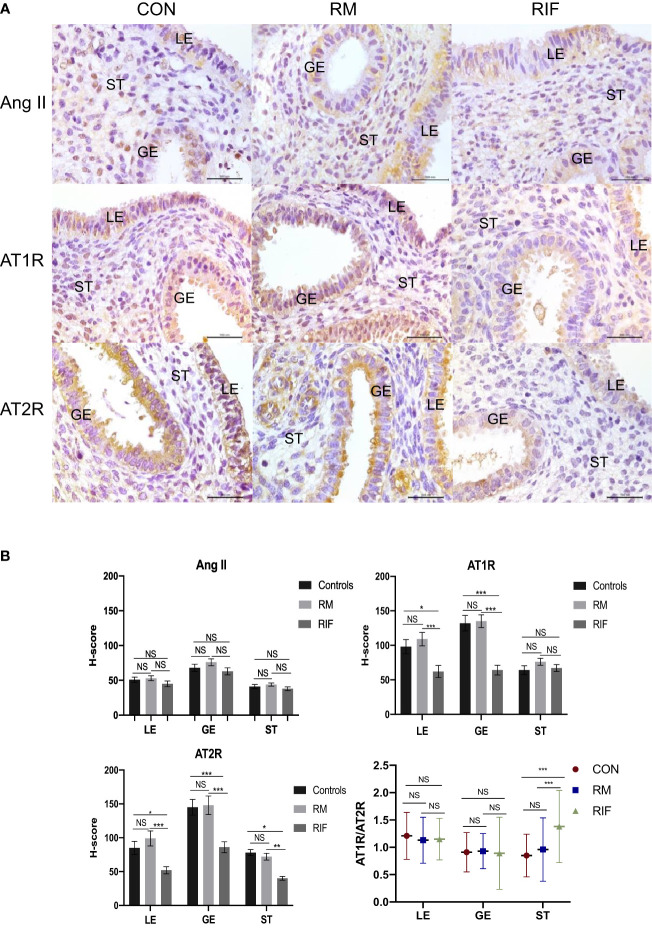
Ang II, AT1R, and AT2R expression in endometrium between fertile controls, RM and RIF. **(A)** Representative images of immunohistochemical staining of Ang II, AT1R, and AT2R among groups. LE, luminal epithelium; GE, glandular epithelium; ST, stroma cells. Scale bar=100 μm. **(B)** Corresponding quantitative analysis. Data are presented as mean ± SEM for AngII, AT1R, and AT2R and median (range) for stromal AT1R/AT2R expression ratios in all compartments of endometrium. *p < 0.05, ***p<0.001, NS, not significant.

There was no significant difference in Ang II expression in either LE, GE, or ST among fertile controls, RM, and RIF groups (**Figure 1A**). When compared with fertile controls, expression of AT1R was significantly lower in LE (38.8% lower) and GE (51.9% lower) only, and expression of AT2R was significantly lower in aII compartments of the endometrium, including LE (37.5% lower), GE (44.1% decreased), and ST (46.7% lower) in RIF groups (**Figure 1B**). While there was no significant difference in the expression of AT1R and AT2R in any compartment between women with RM and the control group. AT1R/AT2R ratio was significantly higher only in the stroma of the RIF group (96.3% higher) when compared with fertile control and RM groups, but there was no significant difference in Ang II, AT1R, AT2R expressions, and AT1R/AT2R expression ratio in stroma between women with RM and control group (**Figure 1B**).

**Figure 2:**
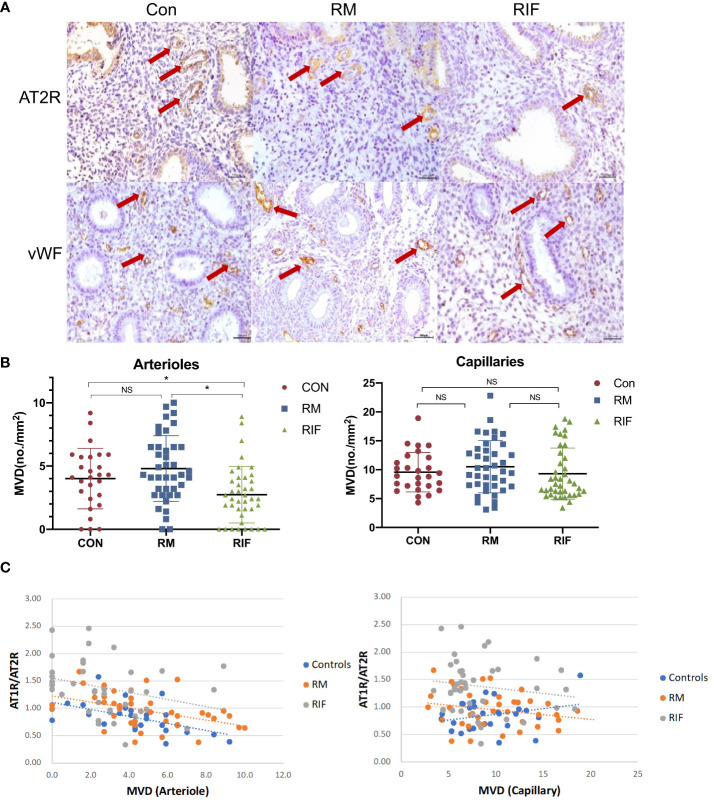
Arteriole and capillary density in endometrium between fertile controls, RM, and RIF. **(A)** Representative images of arterioles stained by AT2R and capillary stained by vWF (arrows) among groups. Scale bar = 100 μm. **(B)** Corresponding quantitative analysis. Data are presented as mean ± SEM. *p<0.05; NS, not significant. **(C)** Correlation analysis of stromal AT1R/AT2R expression ratio with arteriole density in control (r=-0.559, p=0.002), RM (r=-0.437, p=0.001) and RIF (r=-0.419, p=0.007), and its correlation with capillary density in control (r=0.256, p=0.197), RM (r=-0.227, p=0.158), and RIF (r=-0.157, p=0.334).

The endometrium arteriole MVD in the stroma of women with RIF (25.0% decreased) was found to be significantly lower than that in the fertile controls and RM groups, but there was no significant difference between RM and fertile control group (**Figures 2A, B**). The endometrium capillary MVD was not significantly different among fertile controls, RM, and RIF. In addition, endometrium arteriole MVD (r= -0.419, p=0007), but not capillary MVD (r= -0.157, p=0.3334), in the stroma was significantly and negatively correlated with stromal AT1R/AT2R ratio in the endometrium (**Figure 2C**).

### Comparisons between pregnant and non-pregnant women

**Figure 3:**
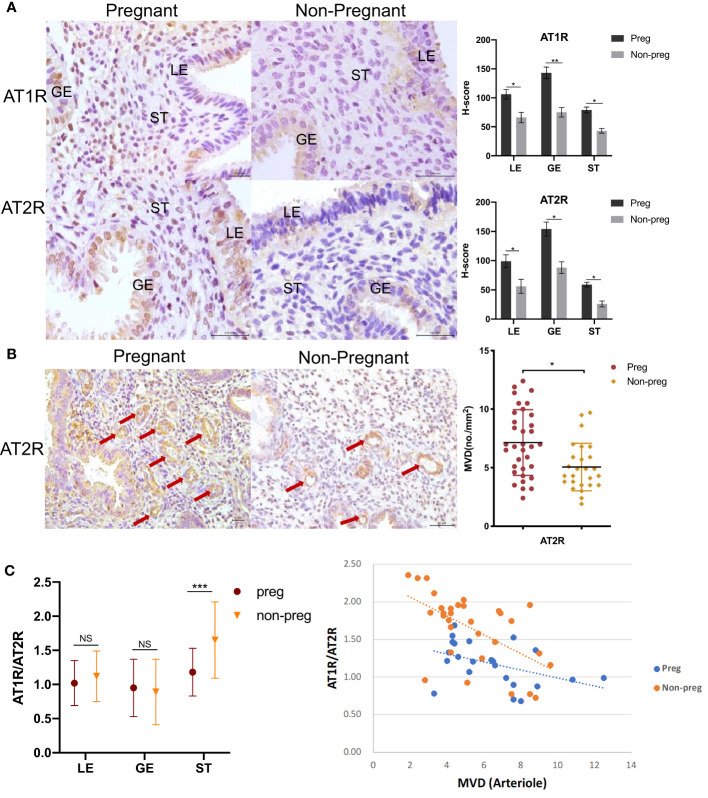
AT1R and AT2R expression in endometrium between women who did (Pregnant) and did not conceive (Non-pregnant) after euploid embryos transfer. **(A)** Representative images of immunohistochemical staining of AT1R and AT2R are shown on the left. LE=luminal epithelium; GE=glandular epithelium; ST=stroma cells. Scale bar=100 μm. Corresponding quantitative results between groups are shown on the right. Data are presented as mean ± SEM. **(B)** Representative images of arterioles stained by AT2R (arrows) are shown on the left. Scale bar=50 μm. Corresponding quantitative analysis between groups is shown on the right. Data are presented as mean ± SEM. **(C)** Comparison of the expression AT1R/AT2R ratio in all compartments of endometrium between the pregnant and non-pregnant groups is shown on the left. Data are presented as median (range). Correlation between stromal AT1R/AT2R ratio with arteriole density in the pregnant group (r=-0.438, p=0.036) and the non-pregnant group (r=-0.577, p=0.001) is shown on the right. *p<0.05, **p<0.01, ***p<0.001, NS, not significant.

There were no significant differences in hormonal profiles, endometrium thickness, and embryo quality (**Table 1**). Expression of both AT1R and AT2R was found significantly lower in all endometrium compartments (AT1R: LE, 41.2 lower; GE, 51.7% lower; ST, 36.6% lower. AT2R: LE, 49.0% lower; GE, 43.3% lower; ST,.54.5% lower) than the non-pregnant group when compared that with the pregnant group (**Figures 3A, B**), and AT1R/AT2R ratio was significantly higher in ST (39.4% higher), but not LE and GE, in the non-pregnancy group than in the pregnancy group (**Figure 3C**). Arteriole MVD was significantly lower in the non-pregnancy group and negatively correlated with stromal AT1R/AT2R ratio (r= -0.577, p=0.001).

## Discussion

This is the first study to compare Ang II and its receptors in the endometrium of fertile control, RM, and RIF during the window of implantation and correlate with the pregnancy outcome after euploid embryo transfer. Our results showed that the expressions of AT1R and AT2R were significantly lower, with AT1R/AT2R ratio significantly higher in RIF and non-pregnancy groups. Additionally, arteriole MVD was significantly lower and negatively correlated with the stromal AT1R/AT2R ratio.

Being known as a cyclical tissue, the endometrium undergoes significant physiological changes of angiogenesis (47). Sufficient angiogenesis has been proven to be a beneficial factor to endometrial receptivity (48, 49), while endometrial vascularity was significantly reduced in women with unexplained subfertility during the mid-late secretory phase regardless of estradiol or progesterone concentrations and endometrial morphometry (50). In our study, differential expression of AT1R and AT2R in peri-implantation endometrium was found between RIF and control groups and between pregnancy and non-pregnancy groups. In addition, two receptors showed similar expression differences in endometrium between RM and RIF, but not between RM and control. It suggests a potential role of local Ang II receptors prior to, not after, embryo implantation. Despite AT1R and AT2R having opposite angiogenic regulatory functions in vessels, with AT1R playing a key role in vasoconstriction and oxidative stress (51, 52), whilst AT2R typically plays an opposing and protective role in such responses (53, 54). The counter-balancing vasomotor responses of AT1R and AT2R have been described (55). From our results, there is a positive correlation between the expressions of two receptors in the endometrium, indicating a synergistic regulation of Ang II receptors in the peri-implantation endometrium. The expressions of both AT1R and AT2R in RIF and non-pregnancy groups were significantly lower but the ratio of AT1R/AT2R was significantly higher compared to fertile women. The degree of the decreased expression of AT2R was more severe than that in AT1R, indicating potentially more vasoconstriction and blood flow restriction. Moreover, the implantation site is surrounded by large-diameter vessels, and the vessels closest to the embryo have more and wider average diameters compared to those further away from the implantation site. The blood flow through these dilated vessels near the implantation site was found to be sluggish, which rarely happens in vessels distant to the embryo (56). Therefore, an appropriate dynamic equilibrium between vasoconstrictors and vasodilators may be a prerequisite for vascular supply in the endometrium during the embryo implantation period, and an imbalance between the two receptors may result in implantation failure.

As from our results, while there is no significant difference in capillary MVD in endometrium between different subgroups of women, whereas there was significantly lower arterial MVD in RIF and non-pregnant women compared to controls. In addition, we found that the expression of stromal AT1R/AT2R and MVD was negatively correlated, which provided us with a deeper understanding of the regulation of angiogenesis in local endometrium. The relatively lower expression of AT1R compared with AT2R in stroma may cause vasodilation and dilated arterials may represent rich blood supply, which is necessary for endometrial receptivity. And the rich blood supply could also be a result of abundant MVD. While a significant negative correlation was observed between stromal AT1R/AT2R and endometrial arterial MVD, we still do not have sufficient evidence to tell the cause and effect between AT1R/AT2R and MVD in the endometrium stroma. The vasodilatation response is reported to be endothelium-dependent as well as NO-mediated (57). The complex regulatory networks mastering RAS include positive regulators, namely Wnt/β-catenin signaling (58, 59), (pro)renin receptor (60, 61), and PGE2/PGE2 receptor EP4 subtype (62); and negative regulators, such as vitamin D receptor, Klotho (63, 64), and a bunch of nuclear receptors including liver X receptor (LXR) (65), peroxisomeproliferator–activated receptors (66), and vitamin D receptor (VDR) (67). Therefore, endothelium cell function and its relationship with the NO signaling pathway in the endometrium angiogenesis and dynamic balance of the regulatory meditators on RAS activity should be further studied.

Conventional angiogenic markers for endometrial angiogenesis mainly focused on the VEGF family and angiopoietins (68, 69). Nevertheless, none of these angiogenic markers could reflect the vasoconstriction and vasodilation status of the vessels. By using AT2R as a surrogate marker of arterioles, not only it can determine the MVD, it may also indicate the arterial vascular tension but further studies are necessary. Small arterioles are responsible for the peripheral resistance in the vasculature, they can regulate blood flow by dilating or constricting the vascular smooth muscle (VSM) tone in response to stimuli (70). Thus by comparing AT1R/AT2R expression ratio, we could have a better understanding of the vascular tone in endometrium between different groups of women. Another strength of this study is that we include a cohort to compare the endometrium between pregnant and non-pregnant women after euploid embryos were transferred, given that embryo factors also contributed greatly to implantation failure (71, 72). Therefore, this cohort is an ideal model to explore implantation failure caused by endometrium factors exclusively without confounding factors from the embryo.

AII the endometrium in this study was collected precisely on the putative day of embryo implantation. Given that RAS was shown to undergo cyclic changes in the endometrium during the menstrual cycle (73, 74), collecting samples at precise timing is critical to our results. Besides, objective quantification through computer-assisted scoring techniques was used in this study to decrease misinterpretation of the results and increase standardization of RAS staining evaluation. However, there were several limitations in our study. First, immunohistochemistry is only semiquantitative, but it allows us to compare the distribution of angiogenetic markers in different endometrial compartments. Differential higher expression of Ang II and AT1R in epithelial cells than stromal cells, and abundant micro-vessels in stroma identified by AT2R expression in endothelium would not be possible using other techniques, such as quantitative real-time polymerase chain reaction or Western blot analysis in the bulk endometrium unless single-cell sequencing. The correlation between RAS expression and hormonal level is lacking as the sampling time of blood and endometrial biopsy was not the same. Moreover, in-depth study is needed to explore their molecular regulation and paracrine function of epithelium with stroma. Larger sample size studies are also needed to confirm whether AT1R and AT2R in endometrium could be used as predictive parameters for implantation outcomes.

In conclusion, expressions of AT1R and AT2R in peri-implantation endometrium were significantly lower and associated with lower arteriole MVD in women with RIF and poor IVF outcomes. The insufficient angiogenesis with the dysregulated RAS in endometrium from women with RIF may be involved in the cross-talk and synchronization between the endometrium and the embryo, consequently leading to poor implantation outcomes.

## Data availability statement

The raw data supporting the conclusions of this article will be made available by the authors, without undue reservation.

## Ethics statement

All procedures followed were in accordance with the ethical standards of the Responsible Committee on Human Experimentation (institutional and national) and with the Helsinki Declaration of 1964 and its later amendments. Informed consent was obtained from all the women who participated in the study. This study was approved by the Joint Chinese University of Hong Kong New Territories East Cluster Clinical Research Ethics Committee (CREC Ref. No. 2014.575) and Taiwan Mackay Memorial Hospital Institutional Review Board Approval of Clinical trial (IRB No. 18MMHIS070e).

## Author contributions

RQ wrote the manuscript, RQ and YZ conducted the study and RQ, and CW analyzed the data, W-JY provided the clinical samples, TZ and JC offered clinical advice, all authors read and approved the manuscript.
